# Clinical Value of Serum Cardiac Troponin I, Trimethylamine N-Oxide (TMAO), and Galectin-3 in Canine Myxomatous Mitral Valve Degeneration: A Preliminary Study

**DOI:** 10.3390/vetsci13040335

**Published:** 2026-03-30

**Authors:** Alessandra Gavazza, Andrea Maggiori, Lucia Biagini, Alessandro Fruganti, Oriol Domenech, Dalida Arletti, Maria Chiara Muollo, Chiara Masci, Giacomo Rossi

**Affiliations:** 1 School of Biosciences and Veterinary Medicine, University of Camerino, 62024 Matelica, MC, Italy; alessandra.gavazza@unicam.it (A.G.); andrea.maggiori@unicam.it (A.M.); alessandro.fruganti@unicam.it (A.F.); dalida.arletti@studenti.unicam.it (D.A.); mariachiara.muollo@unicam.it (M.C.M.); chiara.masci@unicam.it (C.M.); giacomo.rossi@unicam.it (G.R.); 2 Department of Cardiology, Anicura Istituto Veterinario di Novara, Strada Provinciale 9, 28060 Granozzo con Monticello, NO, Italy

**Keywords:** cardiac biomarkers, cardiac troponin I, dog, echocardiography, galectin-3, microbiota, myxomatous mitral valve disease, trimethylamine N-oxide

## Abstract

Myxomatous mitral valve disease (MMVD) is a common cardiac disorder in older small- to medium-sized dogs. Diagnosis and staging are primarily based on echocardiography; however, circulating cardiac biomarkers have gained increasing interest because they are rapid and easily measurable. In this context, the investigators’ aim was to study the clinical value of galectin-3 (Gal-3) and trimethylamine N-oxide (TMAO), in dogs affected by MMVD, in comparison to cardiac troponin I (cTnI), a well-established biomarker of myocardial injury, and echocardiography. Twenty-two dogs were classified as healthy controls or affected by MMVD and staged according to American College of Veterinary Internal Medicine (ACVIM) guidelines. Serum concentrations of Gal-3, TMAO, and cTnI were measured and compared among groups. No significant differences in serum Gal-3 concentration were detected among groups (*p* = 0.955). In contrast, TMAO levels were significantly higher in both asymptomatic and symptomatic dogs compared with healthy controls (both *p* < 0.001). Serum cTnI showed limited sensitivity, with increased values observed only in some symptomatic dogs. Despite study limitations, including small sample size and demographic differences among groups, these preliminary findings suggest that TMAO could be a potential biomarker associated with MMVD development and progression; however, further studies are needed to confirm this association.

## Simple Summary

Older small- to medium-sized dogs are prone to a cardiac disorder known as myxomatous mitral valve disease (MMVD). Veterinarians primarily rely on echocardiography for diagnosis and staging, but this approach requires advanced training and specialised equipment, which may limit its availability in smaller veterinary practices. A valuable and significant adjunct to this approach may be the use of circulating cardiac biomarkers, which are rapid and straightforward to measure. The aim of this study was to investigate the clinical value of galectin-3 (Gal-3) and trimethylamine N-oxide (TMAO) in dogs with MMVD. These were compared to cardiac troponin I (cTnI), a well-known biomarker of heart damage, and echocardiography. Twenty-two dogs were classified as either healthy controls or MMVD-affected and staged according to the American College of Veterinary Internal Medicine (ACVIM) guidelines. Serum concentrations of Gal-3, TMAO and cTnI were measured and compared between groups. No significant differences in Gal- 3 serum concentration were detected among the groups (*p* = 0.955). However, TMAO levels were significantly higher in both asymptomatic and symptomatic dogs than in healthy controls (both *p* < 0.001). According to these preliminary findings, TMAO may be a potential biomarker linked to the development and progression of MMVD and further research is needed to confirm this.

## 1. Introduction

Myxomatous mitral valve disease (MMVD) is the most common cardiac disease in dogs [1]. The disease is age-related [2] and shows a high prevalence in small- to medium-sized dogs [3,4]. MMVD is characterized by a progressive myxomatous degeneration of the mitral valve leaflets, resulting in mitral regurgitation [4]. In advanced stages, this process leads to left atrial and ventricular remodelling, volume overload, and the development of congestive heart failure (CHF) [5]. MMVD is typically associated with a long preclinical period [6], whereas clinical signs occur after the onset of CHF and include a hacking dry cough, dyspnoea, exercise intolerance, and syncope [2]. During these advanced stages of the disease, a progressive slowdown in the return circulation leads to CHF, including in the abdominal organs, particularly the liver and intestine [7]. Abdominal congestion via elevated venous pressure causes intestinal wall edema, increased permeability, and severe intestinal dysbiosis [8]. This congestion, combined with systemic hypoperfusion, creates profound intestinal hypoxia and over-expression of certain factors, such as HIF [9,10]. The change in oxygen concentrations and nutrient stagnation, induced by a progressive reduction in villous absorption, generates a highly specific state of dysbiosis, favouring the overgrowth of some bacterial species and severely reducing the survival of others. Consequently, the gut barrier fails, allowing bacteria to enter the blood, increasing systemic inflammation, and worsening cardiac dysfunction [8].

Clinical management of MMVD is commonly guided by the American College of Veterinary Internal Medicine (ACVIM) guidelines [1], which integrate medical history, clinical examination, thoracic radiography, and echocardiographic assessment for diagnostic and staging purposes. Clinical pathology tests are also recommended for baseline patient assessment and for monitoring during therapy, particularly when drugs such as diuretics and angiotensin-converting enzyme (ACE) inhibitors are administered [1,4,11]. Nevertheless, echocardiography remains the gold standard for identifying and assessing the severity of the disease in dogs [12]; however, it requires advanced training and specialised equipment [11,13], which may limit its availability in smaller veterinary practices and private clinical settings. Furthermore, echocardiography provides limited insight into the underlying pathophysiological processes driving tissue pathology. In this context, cardiac biomarkers have gained increasing interest in veterinary cardiology, as they can be easily quantified in the bloodstream [11] and reflect specific pathophysiological mechanisms related to cardiac injury, often in proportion to the extent of damage [13]. The cardiac biomarkers most extensively investigated in dogs include N-terminal pro-atrial natriuretic peptide (NT-proANP), N-terminal pro-brain natriuretic peptide (NT-proBNP), and cardiac troponin I (cTnI) [14]. NT-proANP and NT-proBNP are released into the circulation in response to atrial and ventricular myocardial stretch due to volume overload [4], whereas cTnI is released due to myocardial cell injury [4,11].

More recently, studies in human medicine have identified galectin-3 (Gal-3) and trimethylamine-N-oxide (TMAO) as promising biomarkers for predicting disease progression and outcomes in cardiac disorders [11,15]. Gal-3, a β-galactoside-binding protein secreted by activated macrophages, has been shown to promote fibroblast activation, which contributes to myocardial fibrosis [16,17]. TMAO is an oxidation product of the gut microbiota-derived metabolite trimethylamine (TMA), which is generated in the liver by flavin-containing monooxygenases (FMOs) [18]. TMAO has been shown to promote inflammatory responses by activating the nucleotide-binding oligomerization domain-like receptor protein 3 (NLRP3) inflammasome, leading to cardiac fibroblast proliferation [19]. In human cardiology, both Gal-3 and TMAO are considered indicators of cardiac fibrosis [17], a key pathological process in the development of heart failure (HF) [16,17].

This study aimed to investigate the clinical reliability of Gal-3, TMAO and cTnI as circulating biomarkers in dogs. This was achieved by evaluating their serum concentrations in healthy dogs and those affected by MMVD and assessing their ability to differentiate between dogs with MMVD at various stages of the disease.

cTnI, a well-established biomarker of myocardial injury, was notably included as a validated reference marker to strengthen the comparative evaluation of Gal-3 and TMAO.

## 2. Materials and Methods

### 2.1. Animals

A total of 23 dogs were included in this case-control study. Dogs were recruited between 2024 and 2025 from four veterinary medical centers: the Veterinary Teaching Hospital of the University of Camerino, AniCura Istituto Veterinario Novara, AniCura Clinica Veterinaria CMV Varese, and Futuravet Veterinary Clinic. Data regarding breed, sex, age, body weight, ongoing medical treatments and feeding management were obtained from clinical records. In all cases, the animals were fed a commercial diet according to their owners’ preferences. Dogs younger than 2 years of age, with a body weight greater than 40 kg, pregnant, or affected by concomitant systemic or cardiac diseases were excluded from the study population. All dogs underwent a complete physical examination and transthoracic echocardiography. After initial evaluation, one dog was excluded from the study population due to the presence of a suspected left ventricular thrombus whose origin was not attributable to MMVD.

Based on physical examination results and echocardiographic findings, and according to the American College of Veterinary Internal Medicine (ACVIM) staging system [1], dogs were stratified into three groups:

• Symptomatic: ACVIM C Stage (n = 8).
• Asymptomatic: ACVIM B1 or B2 Stages (n = 8).
• Controls: healthy subjects (n = 6).

Dogs were also classified according to MINE score 2 [20,21] for an objective echocardiographic evaluation of the severity of MMVD.

### 2.2. Echocardiography

Experienced operators performed transthoracic echocardiographic examinations to diagnose MMVD, assess disease severity and rule out other cardiac conditions. Dogs were examined without sedation and positioned in right and left lateral recumbency. Standard echocardiographic data were obtained using two-dimensional (2D), M-mode, and Doppler techniques. The diagnosis of MMVD was based on the echocardiographic evidence of thickened or prolapsing mitral valve leaflets, as well as the presence of a mitral regurgitant jet. This was assessed using the right parasternal long-axis and left apical four-chamber views. Mitral regurgitation was evaluated using color-flow Doppler imaging, while pulsed-wave Doppler was used to measure the peak velocity of transmitral flow during early (E-wave) and late (A-wave) diastole from the left apical four-chamber view. The ratio of E-wave to A-wave velocities (E/A ratio) was then calculated. Additional echocardiographic variables included the left atrium-to-aorta ratio (LA:Ao), which was obtained from the right parasternal short-axis view [22], and the left ventricular internal diameter in diastole normalised for body weight (LVIDDn), as measured from the right parasternal short-axis view using M-mode [23]. M-mode measurements from the same view were also used to assess left ventricular fractional shortening (FS). Continuous single-lead electrocardiographic monitoring was maintained throughout the echocardiographic examination.

### 2.3. Complete Blood Count and Serum Biochemistry

A Complete Blood Count (CBC) was performed on whole blood samples collected in K-EDTA tubes from dogs that had been fasted for at least eight hours prior to sampling. All samples were analysed within one hour of collection using a ProCyte Dx haematology analyser (IDEXX, Westbrook, Maine, USA)^®^. The analyser provided the following parameters: red blood cell count (RBC), haematocrit (HCT), haemoglobin (HGB), mean corpuscular volume (MCV), mean corpuscular haemoglobin (MCH), mean corpuscular haemoglobin concentration (MCHC), red cell distribution width (RDW), reticulocytes (RETIC; % and #), reticulocyte haemoglobin (Ret-He), nucleated red blood cells (nRBC; when present), white blood cell count (WBC), neutrophils (NEU; % and #), lymphocytes (LYM; % and #), monocytes (MONO; % and #), eosinophils (EOS; % and #), basophils (BASO; % and #), band neutrophils (BAND; when suspected), platelet count (PLT), platelet distribution width (PDW), mean platelet volume (MPV), and plateletcrit (PCT).

Serum biochemistry analyses were performed on serum samples obtained by centrifugation, using a validated automated veterinary chemistry analyser (BT3500VET^®^, Biotecnica Instruments, Rome, Italy), which provided the following parameters: Calcium (Ca), Glucose, Aspartate aminotransferase (AST), Alanine aminotransferase (ALT), Blood urea nitrogen (BUN), Gamma-glutamyl transferase (GGT), Cholesterol, Alkaline phosphatase (ALP), Triglycerides, Albumin, Creatinine, Total protein, Direct bilirubin, Total bilirubin, Indirect bilirubin, Phosphorus, Corrected calcium, Calcium-to-phosphorus ratio (Ca/P ratio), Globulins.

### 2.4. GAL-3, TMAO and cTnI

The concentrations of the serum cardiac biomarkers cTnI, Gal-3, and TMAO were determined using serum samples stored at −20 °C and subsequently transferred to −80 °C until analysis. Stored samples underwent a single freeze–thaw cycle prior to analysis. Serum samples for cTnI analysis were shipped under refrigerated conditions to the CDVet Veterinary Diagnostic Laboratory (Rome, Italy), where they were analysed. According to the laboratory reference range, serum cTnI concentrations above 0.20 ng/L were considered abnormal. Gal-3 and TMAO concentrations (ng/mL) were measured using enzyme-linked immunosorbent assay (ELISA) techniques. Gal-3 concentrations were determined using a canine Gal-3 competitive ELISA kit (BlueGene Biotech, Shanghai, China; catalogue no. E08G0052), while TMAO concentrations were measured using a canine TMAO competitive ELISA kit (MyBioSource, San Diego, CA, USA; catalog no. MBS7271760), according to the manufacturers’ instructions. The same procedure was followed for both assays. Serum samples were allowed to thaw at room temperature for approximately one hour before use. All reagents were stored in accordance with the manufacturers’ instructions and allowed to reach room temperature for approximately one hour before the procedure began. Subsequently, 100 μL of either standards or samples was dispensed into the designated wells, while 100 μL of phosphate-buffered saline (PBS, pH 7.0–7.2) was added to the blank control well. Afterwards, 50 μL of conjugate was added to each well except for the blank control, and the contents were carefully mixed. The microplate was then covered and incubated at 37 °C for one hour, followed by five manual washing steps. Next, 50 μL of substrate solutions A and B were added to each well and the plate was incubated at 37 °C for 15 min. Finally, 50 μL of stop solution was added to terminate the reaction, and the optical density (OD) was measured immediately at 450 nm using a microplate reader (BioTek, Winooski, VT, USA) [24]. As indicated in the technical data sheets provided by the manufacturers, standard curves were generated from the OD values of the standards using a four-parameter logistic (4PL) regression model. Biomarker concentrations were then calculated by interpolation from the corresponding OD values. All samples were tested in duplicate, and the assay was performed twice on the same morning to ensure intra-assay consistency. According to the manufacturers, the intra- and inter-assay coefficients of variation were less than 10% and 12%, respectively, for both analytes, while the sensitivity of the assay was 0.1 ng/mL for galectin-3 (Gal-3) and 1.0 ng/mL for TMAO. No significant cross-reactivity or interference with related analogues has been reported for either assay. However, cross-reactivity with untested compounds cannot be ruled out entirely.

### 2.5. Statistical Analysis

Statistical analyses were performed to compare the following echocardiographic parameters across different ACVIM stages: LA/Ao, LVIDDN, FS%, E-Vel (m/s), A-Vel (m/s) and E/A. Analyses were also conducted to compare circulating Gal-3 and TMAO concentrations in control subjects, asymptomatic subjects (dogs in ACVIM stages B1 and B2), and symptomatic subjects (dogs in ACVIM stage C). The Shapiro–Wilk test was used to assess data distribution within each group. Homogeneity of variances was evaluated using Levene’s test. For Gal-3, as the homogeneity of variances assumption was met, group comparisons were performed using a one-way analysis of variance (one-way ANOVA), followed by a Tukey’s honestly significant difference (HSD) test for post hoc pairwise comparisons. For TMAO, however, the homogeneity of variances assumption was violated, so group differences were assessed using Welch’s one-way ANOVA, with Games–Howell post hoc tests for multiple comparisons. Effect sizes were estimated using omega squared (ω^2^) for the one-way ANOVA model and were interpreted according to conventional thresholds. Continuous variables are reported as mean ± standard deviation (SD), median, and range. The 95% confidence intervals (95% CI) for the mean were calculated under the assumption of a t-distribution. Statistical significance was set at *p* < 0.05. All statistical analyses were performed using Jamovi (version 2.6.44; The Jamovi Project). A Chi-square test of independence was used to assess the association between group membership and cTnI values, and Cramér’s V was calculated as a measure of effect size. A Post hoc analysis using Pearson residuals was then performed to identify which groups contributed most strongly to the observed differences.

## 3. Results

Of the 22 dogs included in the study, 10 were male (4 intact and 6 neutered) and 12 were female (3 intact and 9 spayed). The mean age was 9.7 ± 3.5 years (range, 2–15 years), and the mean body weight was 11.8 ± 9.6 kg (range, 2.6–37.3 kg). Most dogs were mixed-breed (n = 8), followed by Cavalier King Charles Spaniel (n = 3), Belgian Malinois (n = 1), Chesapeake Bay Retriever (n = 1), Chihuahua (n = 1), Dachshund (n = 1), Epagneul Breton (n = 1), French Bulldog (n = 1), German Shepherd Dog (n = 1), Jack Russell Terrier (n = 1), Maltese (n = 1), Miniature Pinscher (n = 1), and Shetland Sheepdog (n = 1). Based on echocardiographic findings and the American College of Veterinary Internal Medicine (ACVIM) staging system [1], 5 dogs were classified as stage B1, 3 as stage B2, and 8 as stage C, while 6 dogs without echocardiographic evidence of MMVD served as healthy controls (Table 1).

All of the dogs in stage C and two of the dogs in stage B2 were on pharmacological treatment when we recruited them. The two-stage B2 dogs were treated with a combination of pimobendan, an ACE inhibitor and spironolactone. One of these dogs was also receiving furosemide for diuretic therapy. All stage C dogs were treated with a combination of pimobendan, an ACE inhibitor, spironolactone and furosemide. One canine with stage C received additional treatment involving the use of torsemide and sildenafil.

Left cardiac chamber enlargement was assessed using the LA/Ao ratio and LVIDDN. There were significant differences in both parameters across ACVIM stages (*p* < 0.001 for both). Post hoc analysis revealed significantly higher LA/Ao values in stage C dogs compared with healthy controls (*p* < 0.001) and stages B1 (*p* < 0.001) and B2 (*p* = 0.009). Similarly, LVIDDN values were significantly higher in stage C dogs than in healthy controls (*p* < 0.001) and stage B1 dogs (*p* = 0.001). Overall, both parameters showed a progressive increase with advancing disease severity, as defined by the ACVIM staging system. Left ventricular systolic function, as assessed by fractional shortening (FS%), also differed significantly across ACVIM stages (*p* = 0.016), with higher values observed in stage C dogs compared with those in healthy controls (*p* = 0.012).

Peak E-wave and A-wave velocities also differed significantly across ACVIM stages (*p* < 0.001 and *p* = 0.005, respectively). Post hoc analysis showed that E-wave velocity was significantly higher in stage C dogs compared with healthy controls (*p* = 0.002), stage B1 (*p* < 0.001), and stage B2 (*p* = 0.043). Similarly, A-wave velocity was significantly higher in stage C dogs compared with healthy controls (*p* = 0.009) and stage B1 dogs (*p* = 0.016). No significant differences in the E/A ratio were observed across ACVIM stages (*p* = 0.118) (Table 2).

In addition, dogs were classified according to the MINE score 2 [20,21] in order to provide an objective echocardiographic assessment of MMVD severity. Overall, twelve dogs were classified as mild (all of the healthy controls and B1 stage dogs, plus one stage B2 dog); one dog was classified as moderate (one stage B2 dog); eight dogs were classified as severe (one stage B2 dog and seven stage C dogs); and one dog was classified as late stage (one stage C dog) (Table 3).

Gal-3 and TMAO levels were compared among controls, asymptomatic subjects, and symptomatic subjects.

Gal-3 was measured in serum samples obtained from all 22 dogs. After measurement, two samples from the asymptomatic group were excluded from statistical analysis because identified as outliers (Table 4).

TMAO concentrations were measured in 19 of the 22 dogs included in the study, due to insufficient serum volume in three samples (Table 5).

**Figure 1:**
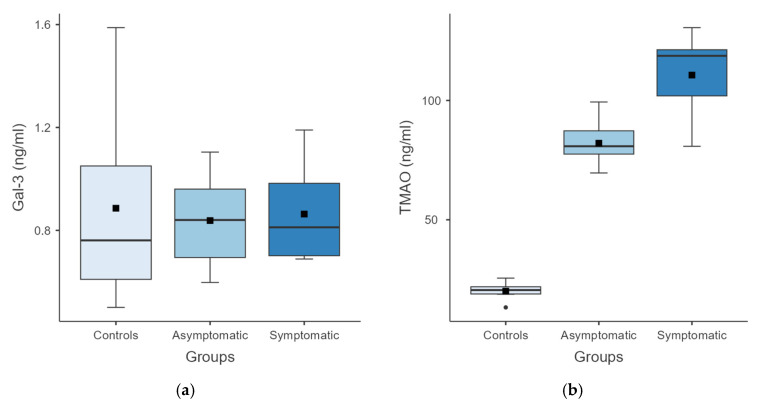
Box plots showing Gal-3 (**a**) and TMAO (**b**) levels in controls, asymptomatic subjects, and symptomatic subjects. Boxes represent the interquartile range, the horizontal line indicates the median, and whiskers indicate minimum and maximum values. For Gal-3, no significant differences were detected among groups (one-way ANOVA, *p* = 0.955). In contrast, for TMAO, Welch’s one-way ANOVA revealed a significant group effect (*p* < 0.001). Post-hoc Games–Howell tests showed significantly higher TMAO levels in both asymptomatic and symptomatic subjects compared with controls, whereas the difference between asymptomatic and symptomatic subjects was not statistically significant (*p* = 0.061).

The one-way ANOVA revealed that Gal-3 levels were not significantly different among the three groups (F(2, 17) = 0.047, *p* = 0.955). Post-hoc comparisons showed that there were no significant differences between any of the pairs (all *p* > 0.95). The negligible effect size (ω^2^ ≈ 0) indicates minimal between-group variability in Gal-3 levels between groups. In contrast, a significant group effect was observed for TMAO (F(2, 7.85) = 156, *p* < 0.001), with post-hoc pairwise comparisons showing significantly higher TMAO levels in both asymptomatic and symptomatic subjects compared with controls (both *p* < 0.001). However, the difference between asymptomatic and symptomatic subjects did not reach statistical significance (*p* = 0.061). The large effect size (ω^2^ = 0.898), indicates significant between-group variability in TMAO levels (Figure 1).

**Figure 2:**
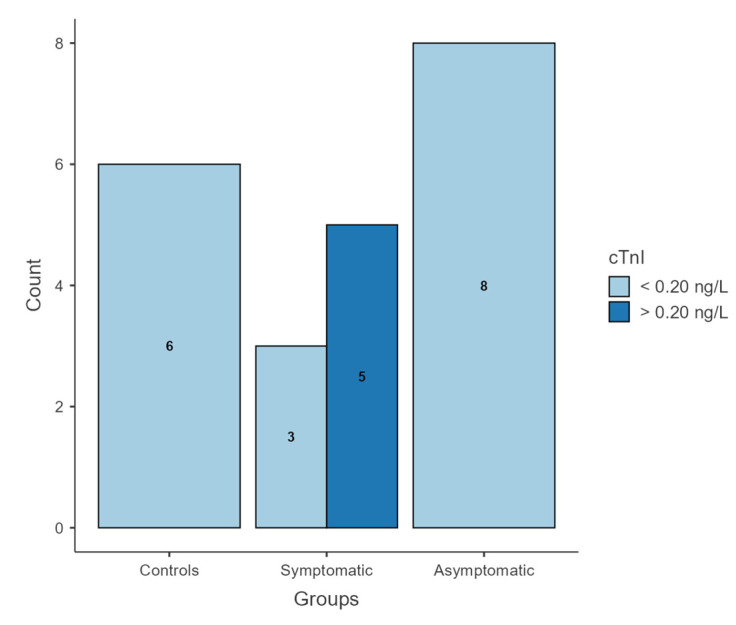
Bar plot showing the distribution of cTnI concentrations across study groups. All dogs included in the control and asymptomatic groups had cTnI concentrations below 0.20 ng/L, whereas elevated cTnI values (>0.20 ng/L) were observed only in a subset of dogs in the symptomatic group.

All dogs included in the asymptomatic and control groups, as well as three dogs in the symptomatic group, showed cTnI concentrations below 0.20 ng/L. In contrast, the remaining five dogs in the symptomatic group showed a cTnI concentration greater than 0.20 ng/L (mean 1.1 ± 0.7 ng/L) (Figure 2).

A chi-square test of independence revealed a statistically significant association between group membership and cTnI concentration (χ^2^(2, N = 22) = 11.3, *p* = 0.003). Values above 0.20 ng/L were exclusively observed in the symptomatic group (5/8), while all dogs in the control and asymptomatic groups had concentrations below 0.20 ng/L. The association exhibited a substantial effect size, as indicated by Cramer’s V (0.717). Post hoc analysis based on Pearson residuals showed that symptomatic dogs with cTnI concentration above 0.20 ng/L contributed most strongly to the observed differences between groups.

## 4. Discussion

The present study set out to evaluate the clinical value of Gal-3, TMAO and cTnI, given their growing relevance in cardiovascular medicine. In this context, we investigated the utility of these serum biomarkers in managing the most common acquired cardiac disease in dogs, namely MMVD [25]. The gold standard technique for the diagnosis, monitoring, and therapeutic management of valvular diseases is represented by echocardiography, as reported in both human and veterinary cardiology [26].

However, studies in human cardiology have demonstrated that a multimodal approach, combining different biomarker measurements with echocardiographic evaluation, is more effective than echocardiographic assessment alone. Indeed, the complementary nature of the information obtained enables a more comprehensive understanding of the disease and promotes an integrated and accurate assessment of the patient [11]. Moreover, in veterinary medicine, blood-based biomarkers offer the practical advantage of easier execution compared with echocardiography [4,11,13]. From this perspective, integrating clinically relevant serum biomarkers for cardiac diseases into routine practice could be highly advantageous, either when echocardiographic evaluation is unavailable or in addition to it. In this preliminary study, 17 dogs affected by MMVD at different stages of severity, together with a control group of 6 healthy dogs, were selected to investigate the clinical utility of Gal-3 and TMAO in relation to an established biomarker such as cTnI, as well as routinely acquired echocardiographic indices. The differentiation of dogs affected by MMVD from those free of disease was achieved through the use of echocardiography, physical examination and clinical history. The classification of affected dogs was then carried out in accordance with the ACVIM guidelines [1]. Given the morpho-functional alterations that primarily influence disease progression, echocardiographic indices related to left chamber enlargement, cardiac function, and intracardiac pressures were evaluated. According to the ACVIM guidelines, left-sided cardiac enlargement is defined by an LA:Ao ratio ≥ 1.6 and an LVIDDn ≥ 1.7. In asymptomatic dogs, exceeding these thresholds constitutes a criterion for distinguishing stage B1 from stage B2 [1].

The LA:Ao ratio clearly demonstrated that left atrial enlargement progressed proportionally with disease severity, confirming its role as a key parameter reflecting the magnitude of mitral regurgitation [27,28]. Ventricular dilation tended to parallel atrial enlargement; however, the differences between disease stages were less pronounced. Overall cardiac function reflects the combined systolic and diastolic performance of the left ventricle, which can be evaluated echocardiographically through FS% and assessment of diastolic filling patterns [29]. Based on the results obtained, however, FS% failed to discriminate among different degrees of mitral disease severity, as a statistically significant difference was observed only between dogs in stage C (advanced disease) and healthy dogs.

The worsening of mitral regurgitation has been linked to an increase in FS%, as indicated by several studies [21], due to its dependence on volume overload [29]. However, this index is influenced by multiple confounding factors, including age, breed, sex, sympathetic tone, and pimobendan therapy [21,30]. Consequently, the interpretation of FS% is challenging and cannot be attributed solely to the underlying disease process. These findings confirm previous evidence suggesting that FS% has limited utility in assessing systolic myocardial function in dogs with MMVD [21,29,31].

Peak E- and A-wave velocities, together with the E:A ratio, allow characterization of left ventricular diastolic filling patterns and may reflect the presence of atrial hypertension [29]. Physiological reference ranges for E- and A-wave velocities are 0.6–0.8 m/s and 0.5–0.6 m/s, respectively [31]. According to the available literature, E-wave velocity is of clinical relevance due to its association with atrial volume and pressure [27,32], and overt atrial hypertension is generally considered when E-wave velocity exceeds 1.5 m/s [29,30].

The current study found that, with the exception of dogs in ACVIM stage C, where both parameters appeared increased, mean E- and A-wave velocities were within normal limits. Nevertheless, E-wave velocity remained below 1.5 m/s even in dogs in stage C. Despite the atrial volume overload suggested by the LA:Ao ratio, atrial pressures did not appear markedly elevated. This discrepancy may be due to the pharmacological therapy administered to all stage C dogs, with previous studies reporting a reduction in E-wave velocity in dogs treated with a combination of benazepril and spironolactone [33], an ACE inhibitor and an aldosterone antagonist, respectively, both of which were included in the therapeutic protocol adopted for all stage C dogs in the present study. Although the E:A ratio physiologically exceeds 1, a marked increase above this value is indicative of atrial hypertension [31]. Such an increase was observed only in stage C dogs, though it did not reach statistical significance.

Since the primary aim of the present study was to evaluate the clinical and prognostic significance of specific serum biomarkers in relation to echocardiography, we applied a staging method based on major echocardiographic indices, namely the MINE score 2 [20,21], to standardise echocardiographic assessment, particularly considering the multicentric nature of the study. While the MINE score 2 primarily holds prognostic value in clinical management, it is based on routinely acquired echocardiographic indices, allowing meaningful correlation with clinical findings. Based on the results obtained across groups, the MINE score 2 proved to be easy to apply and of considerable clinical value, as it showed a progressive increase and a positive correlation with advancing disease stage.

In veterinary medicine, echocardiography allows the detection of morpho-functional alterations involved in the progression of cardiac disease [32] and, consequently, it can facilitate diagnosis, monitoring, and adaptation of therapeutic strategies to the patient’s condition. However, it cannot categorise or quantify the metabolic processes underlying pathological degeneration [11]. Currently recognized biomarkers with established clinical value in veterinary cardiology are mainly NT-proBNP and cTnI [11,14,26]. The former reflects the degree of myocardial stretch, whereas the latter provides information on the structural integrity of myocardial fibers [34]. However, when attempting to define a clinically meaningful biomarker panel, the extent of myocardial inflammation and fibrosis remains difficult to determine [35,36].

In human medicine, myocardial fibrotic degeneration has been recognized as a determinant of heart failure and among the molecules implicated in myocardial fibrotic progression, Gal-3 and TMAO have received increasing attention [16,19,37].

Identifying biomarkers capable of quantifying myocardial fibrosis in dogs with MMVD could therefore represent a highly valuable prognostic tool, as heart failure is the key event in MMVD progression and a major trigger of clinical deterioration [2]. The measurement of Gal-3 and TMAO in the present study aimed to evaluate potential correlations between circulating levels of these molecules and MMVD severity. The objective was to determine whether these biomarkers could reliably reflect disease stage and provide additional clinically useful information. In parallel, quantitative determination of cTnI was performed to assess potential correlations between novel biomarkers and an established marker. Consistent with previous reports, cTnI showed a positive association with disease progression. However, as previously described [4,13], its ability to distinguish between asymptomatic and symptomatic stages appeared limited. Increased cTnI concentrations were observed only in symptomatic dogs (ACVIM stage C), whereas no significant elevation was detected in asymptomatic MMVD dogs (ACVIM stages B1 and B2). Moreover, only five dogs with advanced disease (ACVIM stage C) exhibited cTnI values above the laboratory reference range. These findings highlight the limited sensitivity of cTnI in identifying cardiac remodelling associated with MMVD, particularly in earlier disease stages, and raise concerns regarding its diagnostic sensitivity as a standalone cardiological biomarker. Regarding Gal-3, no significant differences were observed among the groups. This finding is consistent with previous reports showing no significant variation in circulating Gal-3 levels across different stages of mitral insufficiency [24,35,36], although some studies have described differences between healthy and MMVD dogs, which were not observed in the present study [11].

Gal-3 is considered a marker of myocardial fibrosis and is functionally associated primarily with diastolic dysfunction [38]. Fibrotic myocardial degeneration reduces myocardial elasticity and impairs ventricular compliance during rapid filling [24]. Considering that E-wave velocity may have been reduced by therapy with benazepril and spironolactone, it is reasonable to hypothesize that pharmacological treatment may also have influenced circulating Gal-3 concentrations. Indeed, the potential impact of therapy on Gal-3 levels has been previously suggested [24], based on earlier evidence demonstrating that aldosterone antagonist therapy reduces circulating markers of myocardial fibrosis [38].

In contrast to Gal-3 and cTnI, TMAO yielded noteworthy findings and exhibited a trend opposite to that observed for cTnI. Whereas cTnI demonstrated limited sensitivity, with increased concentrations observed only in symptomatic dogs (ACVIM stage C), TMAO concentrations were significantly higher than those of controls in both asymptomatic and symptomatic dogs with MMVD. These findings partially confirm previous reports describing significant differences in TMAO levels between healthy dogs and dogs with symptomatic MMVD [25]. However, in contrast to those reports, no significant differences were detected in the present study between asymptomatic and symptomatic dogs with MMVD.

## 5. Conclusions

Serum cTnI concentrations showed a positive correlation with disease progression but demonstrated limited sensitivity in discriminating between the different stages of MMVD. In contrast to findings reported in human cardiology, the present study showed that serum Gal-3 concentrations did not differ significantly among the analysed groups. More noteworthy findings emerged from the evaluation of TMAO, with serum concentrations significantly higher in dogs with MMVD than in healthy controls, regardless of disease severity. However, no significant differences were observed between symptomatic and asymptomatic dogs. Several limitations of this study should be acknowledged, including the critically small sample size and intergroup variability in breed, body weight, age, and treatment strategies, all of which may have limited the ability to draw robust conclusions. Further research involving larger sample sizes and a more uniform study population in terms of age and body weight may help clarify whether serum TMAO concentrations differ across stages of MMVD. Despite these important limitations, the present findings may be indicative of a possible association between increased TMAO levels and disease progression. Prospective longitudinal studies are warranted to better define the potential prognostic value of TMAO in canine MMVD. Whether increased TMAO production reflects primary gut dysbiosis in cardiac patients or is secondary to gastrointestinal hypoxia remains to be clarified. Resolving this issue could lay the groundwork for future research into microbiota-targeted therapeutic strategies, such as probiotic and nutraceutical interventions aimed at modulating the intestinal microbiota of dogs with MMVD, with the goal of potentially slowing disease progression.

## Figures and Tables

**Table 1:** Demographic characteristics of dogs enrolled in the study grouped by ACVIM stage of MMVD.

		Controls (n = 6) ^a^	B1 (n = 5) ^a^	B2 (n = 3) ^a^	C (n = 8) ^a^
**Breed**	Belgian Malinois			x	
	Cavalier King Charles Spaniel		xx	x	
	Chesapeake Bay Retriever	x			
	Chihuahua				x
	Dachshund	x			
	Epagneul Breton Spaniel				x
	French Bulldog	x			
	German Shepherd Dog	x			
	Jack Russell Terrier			x	
	Maltese				x
	Miniature Pinscher				x
	Mixed-breed	xx	xx		xxxx
	Shetland Sheepdog		x		
**Sex ^b^**	F	x			xx
	M	x		x	xx
	SF	xx	xxx	x	xxx
	NM	xx	xx	x	x
**Age ^c^**		6.4 (±2.9)	9 (±2.3)	8.9 (±2.5)	12.8 (±2.3)
**Weight ^c^**		19.8 (±12.6)	10.4 (±5.7)	13.5 (±11.3)	6.5 (±4.9)

x: corresponds to one dog, repeated symbols indicate the total count. ^a^ number of dogs per ACVIM stage. ^b^ F: Intact female; M: Intact male; SF: Spayed female; NM: Neutered male. ^c^ mean (±SD).

**Table 2:** Acquired echocardiographic measurements (mean ± SD) grouped by ACVIM stages.

	Controls	B1	B2	C
LA/Ao	1.31 (±0.15)	1.47 (±0.17)	1.74 (±0.22)	2.69 (±0.58)
LVIDDN	1.43 (±0.12)	1.38 (±0.22)	1.90 (±0.38)	2.19 (±0.13)
FS%	30.70% (±6.15)	38.20% (±12.48)	34.00% (±9.64)	47.30% (±7.36)
E-Vel (m/s)	0.69 (±0.20)	0.60 (±0.17)	0.82 (±0.27)	1.37 (±0.37)
A-Vel (m/s)	0.56 (±0.13)	0.56 (±0.12)	0.67 (±0.12)	0.85 (±0.18)
E/A	1.24 (±0.07)	1.06 (±0.15)	1.19 (±0.20)	1.72 (±0.70)

*p*-values were obtained using one-way ANOVA: LA/Ao (*p* < 0.001), LVIDDN (*p* < 0.001), FS% (*p* = 0.016), E-Vel (*p* < 0.001), A-Vel (*p* = 0.005), E/A (*p* = 0.118).

**Table 3:** Echocardiographic measurements (mean ± SD) grouped by MINE score 2 stages.

	Mild	Moderate	Severe	Late Stage
LA/Ao	1.40 (±0.17)	1.71 (±0.00)	2.62 (±0.63)	2.51 (±0.00)
LVIDDN	1.42 (±0.16)	1.80 (±0.00)	2.18 (±0.11)	2.42 (±0.00)
FS%	33.75% (±9.45)	27.00% (±0.00)	45.88% (±6.47)	56.00% (±0.00)
E-Vel (m/s)	0.66 (±0.18)	0.57 (±0.00)	1.30 (±0.36)	1.69 (±0.00)
A-Vel (m/s)	0.57 (±0.12)	0.55 (±0.00)	0.84 (±0.18)	0.87 (±0.00)
E/A	1.15 (±0.14)	1.04 (±0.00)	1.66 (±0.70)	1.94 (±0.00)

**Table 4:** Gal-3 levels across study groups.

Group	N	Mean ^a^	95% CI (Mean)	Median ^a^	SD	Min–Max ^a^
Controls	6	0.89	0.46–1.31	0.76	0.41	0.50–1.59
Asymptomatics ^b^	6	0.84	0.64–1.04	0.84	0.19	0.60–1.10
Symptomatics ^c^	8	0.86	0.71–1.02	0.81	0.19	0.69–1.19

Values are reported as mean ± standard deviation; 95% confidence intervals of the mean were computed assuming a t-distribution with N − 1 degrees of freedom. *p*-value obtained using one-way ANOVA (*p* = 0.955). ^a^ ng/mL. ^b^ ACVIM B1 and B2 stages. ^c^ ACVIM C stage.

**Table 5:** TMAO levels across study groups.

Group	N	Mean ^a^	95% CI (Mean)	Median ^a^	SD	Min–Max ^a^
Controls	6	20.1	15.8–24.4	20.5	4.1	13.3–25.5
Asymptomatics ^b^	8	82.1	73.9–90.3	80.8	9.8	69.6–99.4
Symptomatics ^c^	5	111.0	86.3–135	119.0	19.6	80.8–131.0

Values are reported as mean ± standard deviation; 95% confidence intervals of the mean were computed assuming a t-distribution with N − 1 degrees of freedom. *p*-value obtained using one-way ANOVA (*p* < 0.001). ^a^ ng/mL. ^b^ ACVIM B1 and B2 stages. ^c^ ACVIM C stage.

## Data Availability

The original contributions presented in this study are included in the article. Further inquiries can be directed to the corresponding authors.
